# Accuracy of Markerless 3D Motion Capture Evaluation to Differentiate between On/Off Status in Parkinson's Disease after Deep Brain Stimulation

**DOI:** 10.1155/2018/5830364

**Published:** 2018-09-27

**Authors:** Hector R. Martinez, Alexis Garcia-Sarreon, Carlos Camara-Lemarroy, Fortino Salazar, María L. Guerrero-González

**Affiliations:** Tecnologico de Monterrey, Escuela de Medicina y Ciencias de la Salud, Ave. Morones Prieto 3000, 64710 Monterrey, NL, Mexico

## Abstract

**Background:**

Body motion evaluation (BME) by markerless systems is increasingly being considered as an alternative to traditional marker-based technology because they are faster, simpler, and less expensive. They are increasingly used in clinical settings in patients with movement disorders; however, the wide variety of systems available makes results conflicting.

**Research Question:**

The objective of this study was to determine whether a markerless 3D motion capture system is a useful instrument to objectively differentiate between PD patients with DBS in On and Off states and controls and its correlation with the evaluation by means of MDS-UPDRS.

**Methods:**

Six PD patients who underwent deep brain stimulation (DBS) bilaterally in the subthalamic nucleus were evaluated using BME and the Unified Parkinson's Disease Rating Scale (UPDRS-III) with DBS turned On and Off. BME of 16 different movements in six controls paired by age and sex was compared with that in PD patients with DBS in On and Off states.

**Results:**

A better performance in the BME was correlated with a lower UPDRS-III score. There was no statistically significant difference between patients in Off and On states of DBS regarding BME. However, some items such as left shoulder flexion (*p*=0.038), right shoulder rotation (*p*=0.011), and left trunk rotation (*p*=0.023) were different between Off patients and healthy controls.

**Significance:**

Kinematic data obtained with this markerless system could contribute to discriminate between PD patients and healthy controls. This emerging technology may help to clinically evaluate PD patients more objectively.

## 1. Introduction

Three-dimensional (3D) markerless motion capture systems are continuously being considered as an alternative to traditional sensor-based systems in analyzing human movement kinematics and gait [1–8]. Markerless systems are being used increasingly for several reasons. First, traditional systems are time-consuming because they require the placement of several sensors as well as calibration prior to testing (which altogether can take up to 1 hour). In addition, thanks to this rapidness, technicians may scan a greater number of people per day and therefore allowing this study to be at a more reasonable price (in our hospital, the most expensive study is $80 USD while marker-based evaluation may cost around $500 USD).

Lastly, marker-based systems are susceptible to marker placement error, meaning a sensor can be accidentally placed in a different location in test-retest; therefore, reliability is low and operator dependent, which may cause conflicting results [4]. Markerless motion capture systems use light reflection from the skin or specific colored clothes to capture the patient's avatar, and they have been proven to be more consistent when the test is repeated [4, 9].

The markerless system we use in this study is called DARI (Dynamic Athletic Research Institute) (Motion Platform, version 3.2-Denali from Scientific Analytics Inc., Kansas City, KS, USA). The DARI system generates a full-body motion capture skeleton from cloud voxels, which translate to a human's volumetric silhouette that generates the parameters. Using consistent clothing, the algorithms that generate the skeleton also do it consistently. Rosengarden et al. assessed the repeatability of this system by tracking the change in skeleton segment lengths between sessions, totaling 480 sessions, and 9,120 bone segments. He found that the bone length change for a person between sessions was 1.02% with a variance of 0.002 mm and a 99% confidence interval of 0.81 mm [5]. In another study, Perrott concluded that when comparing marker-based versus markerless evaluations, they both report statistically similar ranges of change in the angle of squats in pelvis and lower limbs [9]. These findings illustrate that a 3D markerless motion capture is a highly repeatable system which can have a variety of new applications for athletic and healthcare settings [3–7, 9]. However, there is currently no gold-standard for motion capture systems and the results are not always consistent because they vary from device to device.

Also, traditional marker-based analysis of a single human motion may require days of intensive data compiling and error-correction. By the time the data is ready, the window of application has passed. Markerless systems usually process data faster; however, DARI's software is an advanced tool that consists of a powerful cloud processing software engine that takes thousands of data points comprising each motion and processes them in under a second. An interesting study conducted by Ceseracciu et al. compared markerless and marker-based motion capture technologies by recording the same movement simultaneously. They found that the markerless 3D motion capture was not as accurate as marker-based. However, because of setup compromise, they only used six cameras for the markerless system (instead of eight that the system asked for) [1].

Most of the published studies that use the DARI system and other markerless systems are applied in the field of sports medicine, for professional athletes, rehabilitation, and injury prevention [4]. However, one of the most affordable ones, the Microsoft Kinect has been used in a number of studies to analyze gait and movement disorders. Galna et al. found that the Microsoft Kinect can accurately measure timing and gross spatial characteristics of relevant PD movements, but smaller movements (e.g., hand clasping) did not have the same spatial accuracy [10]. Another study by Bovonsunthonchai examined gait initiation in patients with PD using a force distribution measurement platform and was successful in documenting specific gait characteristics in PD [11]. A similar study but with a different objective was conducted by Ferrarin et al.; they used a marker-based system to examine the effects of subthalamic stimulation on gait kinematics and kinetics in PD, and they were able to find clinical differences in the effects produced by medication (L-dopa) and DBS [12]. Although none of these studies used exactly the same system we did, they describe high accuracy rate in discriminating between PD patients in the DBS On and DBS Off states. To date, there are no published studies using DARI motion capture technology in neurological diseases, including PD. Based on the hardware and software characteristics described, we believe DARI will show even higher accuracy rate than other markerless systems previously used.

The validity and test-retest reliability in a Parkinson's disease patient are complicated to acquire due the heterogeneity of its clinical presentation. This clinical variability is not only present from patient to patient but also may even change several times a day on the same patient, even after medical or surgical treatment. For this reason, we recruited patients that had previously undergone DBS implantation, this made it possible for a single patient to become its own control in On/Off status. Also, in order to have a baseline to which we could compare the data obtained, we recruited healthy patients matched by age and gender. The objective of this study was to determine if a markerless 3D motion capture system is a useful instrument to objectively differentiate between PD patients with DBS in On and Off states and healthy control subjects.

## 2. Methodology

### 2.1. Patients

Six patients with PD, diagnosed in accordance with the UK Parkinson's Disease Society Brain Bank Clinical Diagnostic Criteria by a certified neurologist and movement disorder specialist, were included (Table 1). This is a pilot study, and the sample size was chosen by convenience for accessibility and proximity to the researchers. Inclusion criteria: diagnosis of PD submitted to subthalamic DBS implantation a minimum of 3 months prior to the evaluation, and age limits were 40 to 80 years old. Exclusion criteria: patients with physical disability (i.e., wheelchair, cane, and assistance to daily living activities), history of stroke and physical disability, another neurological disorder other than PD, recent head and limb trauma that limits movement, and treatment with antipsychotics drug or recent botulinum toxin treatment. The patients had undergone bilateral subthalamic nucleus DBS implantation with a mean of 15 months (range, 5–46 months) prior to this evaluation due to motor fluctuations and dyskinesia after more than 3 years of good response to levodopa. The stimulation parameters were optimized to obtain the best clinical response (140–160 Hz, pulse width 60–90 *μ*s, 2.5–3 V). Stimuli were delivered through the deepest electrode contact 0 (−) and1 (+) in a bipolar configuration. In addition, six healthy subjects matched by age and sex were included as controls. The study was approved by our institutional research board, and the patients and controls provided written consent. In the months preceding the test, the PD patients were clinically evaluated by the same physician according to the Unified Parkinson Disease Rating Scale or motor section (UPDRS-III).

### 2.2. Instrument

Actually, there are many markerless motion capture systems in the market, with a broad range of prices, as well as a broad range of reliability. However, the DARI system has been proven to be one of the best for numerous reasons. This system requires a quick calibration at the beginning of each day that the technician can complete in less than 10 minutes. It does not have to be repeated until the following day, no matter how many patients are evaluated. The system depends on a computer-based software that acquires the patient's skeleton or avatar using eighteen high-speed cameras (120 Hz) placed around the room to collect whole body data and delivers kinematic analysis almost instantly using sophisticated biomechanical algorithms.

Also, traditional motion labs use cumbersome floor-mounted pressure plates to measure the forces generated by the body. These require frequent calibration and restrict the subject's movement to a limited area. The DARI's kinetic capture system does not require force plates and can measure joint torques, ground reaction forces, and other measurements without restricting the subject's natural movement [3].

**Figure 1:**
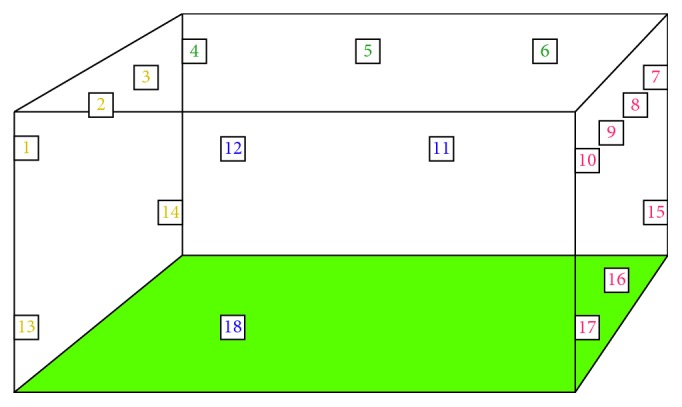
Approximate sketch of BME room, which measured 6 × 6 × 3 meters. Each number represents a camera (18 in total), the different colors of the cameras represent the wall they were on. Cameras were located at two different levels: upper level cameras 1–12 were at a height of 2.6 meters and lower level cameras 13–18 at a height of 30 centimeters from the floor approximately. Distance between upper-level cameras varied but averaged 1.8 meters (range 1.4–2 meters). Distance between lower-level cameras was too variable. The floor was covered in a green screen. Subjects were able to use the entire room for evaluation.

Markerless 3D motion capture evaluation of kinematics in the PD patients and controls was performed in a rectangle room that measures 6 x 6 meters and 3 meters in height. The room has a green screen on the floor, and eighteen cameras are strategically placed on the walls: twelve are placed 2.6 meters high, and 6 are on a lower level at 30 centimeters from the ground. The room has ample space, which allows for broader movements to be analyzed (Figure 1).

### 2.3. Evaluation

PD patients were asked to arrive in the morning wearing dark close-fitting clothing, to skip their last PD medication, and with DBS in the Off state for least 180 minutes. On PD patients, UPDRS-III evaluation was done first. Then, to begin the markerless body motion evaluation (BME), patients and controls' weight and height were entered into the system to help establish the locations of joint centers. Once inside the green room, subjects first stood with feet apart and arms outstretched to the side, while the system created a 3D silhouette of each participant's form and a biometric skeleton was acquired; this took no more than three seconds. For the BME, all subjects performed 16 different movements (Table 2). This set of movements was especially designed to evaluate PD patients and contains items that are related to three major motor symptoms in this disease: rigidity, bradykinesia, and postural instability; tremor is not possible to assess. Once the BME was done, PD patients were asked to turn their DBS to the On state and wait 30 minutes before repeating both UPDRS-III and BME. Controls only performed the BME, which took no more than 20 minutes. PD patients were evaluated twice (DBS states On and Off) with a 30-minute wait in between; their evaluation altogether took approximately 1 hour. The data files were uploaded to DARI Motion Platform where the biomechanical analysis produced full-body kinematic results, and finally, these data were exported to Excel for statistical analysis.

### 2.4. Analysis

A paired *t*-test was used to compare mean changes in UPDRS-III between the On and Off states. Mean differences between groups were evaluated with ANOVA or Kruskal-Wallis tests depending on the distribution of the data of each independent variable. Post hoc analyses were made for pairwise comparison in statistically significant results. Bivariate correlations among evaluation modalities were examined. These correlations were examined in the On and Off states between UPDRS-III and BME items. To compare them as accurately as possible, the items on UPDRS-III and BME that were similar were correlated (e.g., rigidity in upper limbs from UPDRS-III was correlated with shoulder flexion, extension, and rotation from BME). One of the correlations was hip displacement taken from BME, which analyses balance by measuring the movement of hips when patients stand during 10 seconds with arms outstretched to the sides and eyes closed; this was correlated with the posture stability item from UPDRS-III, which is a quick pull, reactionary intervention test where patient's response is measured. Because not all UPDRS-III items were measurable by DARI, seven out of 18 were correlated; however, all BME items were correlated with the UPDRS-III global score (Table 2). IBM SPSS Statistics 21.0 software was used for data analysis. A *p* value of ≤ 0.05 was considered to indicate statistical significance, and values are given as means ± SD.

## 3. Results

Six patients with PD (four men and two women), with a mean age of 62.3 ± 10.6 years (range, 44–76 years), and six control subjects (four men and two women), with a mean age of 60.3 ± 10.25 years (range, 44–76 years), were included in the study.

### 3.1. Differences between DBS in On and Off States

The UPDRS-III motor score differed significantly between PD patients in the DBS On and Off states (30.17 vs. 46.33, *p*=0.028). Among upper limb parameters, we found statistically significant group differences between left shoulder flexion and right shoulder rotation (*p*=0.039  and  0.007, respectively), which in the post hoc pairwise analysis showed an important difference between Off and controls for the left shoulder flexion (*p*=0.038) and for Off and On groups compared with controls for right shoulder rotation (*p*=0.011  and  0.030, respectively) (Table 3).

Regarding trunk mobility, flexion/extension displacement, and right and left rotation showed significant differences between the multigroup analysis (*p*=0.046, 0.049,  0.021, and  0.025, respectively). However, paired differences were just found between On and controls for the right rotation of the trunk (*p*=0.024) and between Off and controls for the left rotation (*p*=0.023). Neither the lower limb nor gait parameters showed statistically significant difference in the multi group analysis (Table 3).

### 3.2. Correlation Extra Gait Items

In UPDRS-III, a higher number indicates a worse performance; in BME, a higher number usually indicates a better performance. A significant negative correlation between the BME and UPDRS-III scores for an item would mean that the item is potentially useful for evaluating patients with PD. Among PD patients in the DBS Off state, significant negative correlations were found between the global UPDRS-III motor score and right shoulder flexion (*r*=−0.829, *p*=0.042) and maximal abduction (*r*=−0.833, *p*=0.039). Right upper extremity rigidity also was negatively correlated with right shoulder extension (*r*=−0.878, *p*=0.021). Negative correlations between UPDRS-III and BME were not present in PD patients in the DBS On state. With regard to the lower extremities, greater depth of bilateral squat was negatively correlated with the UPDRS-III items gait (*r*=−0.926, *p*=0.008) and body bradykinesia (*r*=−0.926, *p*=0.008) in the DBS Off state. This negative correlation was not present in PD patients in the DBS On state. Interestingly, when a PD patient was under stimulation, the performance of a complicated task, such as the displacement of a lunge, was correlated with lower scores for the same items (gait and bradykinesia) in the UPDRS-III (*r*=−0.845, *p*=0.034 for both items).

With regard to trunk evaluation, there was a significant negative correlation when the PD patient was in the DBS On state for the global motor score in UPDRS-III and right (*r*=−0.928, *p*=0.008) and left (*r*=−0.829, *p*=0.048) trunk rotation in BME. Balance and posture showed no significant differences between UPDRS-III and BME (Table 4).

### 3.3. Correlation Gait Items

In PD patients in the DBS Off state, there was a significant negative correlation between UPDRS-III gait items and stride length (*r*=−0.833, *p*=0.039) as well as right step length (*r*=−0.926, *p*=0.008). In our sample, step width in the right and left feet was highly positively correlated with the gait item in UPDRS-III (*r*=−0.926, *p*=0.008 for both feet). Interestingly, cadence, gait speed, and stride length were negatively correlated with the “rising from a chair” item of UPDRS-III (*r*=−0.878, *p*=0.021). In PD patients in the DBS On state, posture was the only UPDRS-III item that was significantly negatively correlated with cadence and gait speed (*r*=−0.926, *p*=0.008 for both items) and with stride length and step length on both sides (*r*=−0.833, *p*=0.039) (Table 4).

## 4. Limitations

We acknowledge the limitations that may interfere with our results. The sample size was small and may not reflect the patterns of all PD patients with DBS; however, this is a pilot study, and sample size was chosen by convenience. Items from UPDRS-III and BME are not the same and therefore, even though correlations were made based on similarity, they may not be perfectly comparable; in UPDRS-III gait, analysis is performed in 9 meters distance, while gait analysis in BME is done in a 6-meter room. However, we hypothesized that BME would have given us an objective quantification of the motor abnormalities, in contrast to the inter-observer variability that may result in UPDRS-III. Also, UPDRS-III includes 18 items, and only seven of them were identifiable by the motion capture system, and the rest were not included; however, global UPDRS-III score was compared to all BME items.

## 5. Discussion

In the present study, we showed that a markerless 3D system correlated with UPDRS-III scores. The utility of markerless techniques in the evaluation of movement disorders in a clinical setting is controversial because analysis parameters have not been standardized yet. However, several studies have described precise acquisition of human anatomy and consistency with recommendations of biomechanical societies [1, 3, 5–7, 9, 10].

Different markerless systems have also been evaluated, including the Microsoft Kinect sensor, which accurately measures timing and gross spatial characteristics of clinically relevant movements but is much less effective for evaluating fine movements, such as tremor, hand clasping, and toe tapping [10, 13]. Because there is a wide variety of markerless systems, it is essential to use a system that is validated and can show repeatability [14]. Even though the DARI system has not been used for neurological movement disorders, including PD, we believe that the previous publications, which acquired data from healthy subjects, confirm its validity [4].

In our study, there was a significant negative correlation between gait and stride length in the UPDRS-III and BME (*r*=−0.833, *p*=0.039) as well as right step length (*r*=−0.926, *p*=0.008) in the Off state. Improvement in some features in the BME was associated with positive changes in the UPDRS-III motor score. Some items showed significance on one side but not the other, this may be due to the asymmetrical clinical course that is typically observed in PD. Although our initial hypothesis of the ability of the DARI system to discriminate between Off and On states of DBS did not meet our expectations, our results suggest a potential use of the DARI system in PD. It is possible that the small sample size used in this pilot study may have reflected statistical results that are not representative. A greater sample size would contribute to establish parameters and give rise to new tools for the objective evaluation of motor disorders; this way, avoiding inter-rater variability. Validation of markerless systems for evaluating patients with PD will require further study protocols involving a greater number of patients in different clinical conditions. There is a wide area of opportunity in the evaluation of movement disorders using emerging technology. Prospective studies would also be useful to establish the clinical significance of markerless systems.

## Figures and Tables

**Table 1:** Parkinson patients at the time of evaluation.

Patient	Age	Time since diagnosis	Time since DBS implantation	PD medication	UPDRS score Off	UPDRS score On
1	67	5 years	10 months	L-dopa, rotigotine	47	22
2	65	12 years	5 months	L-dopa, rotigotine, amantadine	53	27
3	44	5 years	6 months	L-dopa, biperidone	39	29
4	76	10 years	12 months	L-dopa, rotigotine, amantadine	42	37
5	59	5 years	11 months	L-dopa, rotigotine	57	41
6	63	10 years	46 months	L-dopa, rotigotine, rasagiline	40	25

PD medication doses: L-dopa ranged from 400 to 1000 mg per day, rotigotine 4–8 mg per day, amantadine ranged from 100 to 300 mg per day, and rasagiline 1 mg per day.

**Table 2:** Comparable items between the UPDRS-III and Body Motion Evaluation.

Functional modalities	UPDRS-III items	BME items	Description of BME items
Upper extremity	Rigidity (upper limbs 3.3)	Shoulder flexion (right and left)	All are range of motion, measured in degrees
	Body bradykinesia (3.14)	Shoulder extension (right and left)	
		Shoulder internal/external rotation (right and left)	
		Maximum shoulder abduction (right and left)	

Lower extremity	Rigidity (lower limbs 3.3)	Bilateral squat depth	All measured in centimeters and inches
	Gait (3.10)	Lunge distance (right and left)	
	Body bradykinesia (3.14)		

Trunk	Rigidity (neck 3.3)	Trunk rotation (right and left)	All measured in degrees
	Posture (3.13)	Trunk flexion	
		Trunk extension	

Balance and posture	Postural stability (3.12)	Anterior-posterior hip displacement	Patients were asked to remain for 10 seconds with arms outstretched to the sides, eyes closed, and head tilted upward
	Posture (3.13)	Medial-lateral hip displacement	Hip displacement was measured in centimeters

Gait	Arising from a chair (3.9)	Cadence	Strides/minute
	Gait (3.10)	Gait speed	Meters/second
	Freezing of gait (3.11)	Stride length	Centimeters
	Posture (3.13)	Step length (right and left)	Centimeters
	Body bradykinesia (3.14)	Step width (right and left)	Centimeters

BME, Body Motion Evaluation; UPDRS-III, Unified Parkinson's Disease Rating Scale.

**Table 3:** Mean ± SD differences in items between patients in DBS On, DBS Off, and controls.

Extra gait items				*p* value mean difference between groups/pos hoc pairwise comparison that showed significance
Items	DBS Off	DBS On	Controls	
Shoulder flexion (°)	R: 164.01 ± 13.41	R: 166.91 ± 20.82	R: 183.43 ± 14.12	NS
	**L: 160.00** **±** **15.23**	L: 165.05 ± 16.50	**L: 183.48** **±** **12.13**	0.039/**0.038**
Shoulder rotation (°)^*∗*^	**R: 147.33** **±** **18.94**	R: 152.60 ± 20.20	**R: 184.53** **±** **16.91**	0.07/**0.011**
	R: 147.33 ± 18.94	**R: 152.60** **±** **20.20**	**R: 184.53** **±** **16.91**	0.07/**0.030**
	L: 141.41 ± 16.76	L: 142.11 ± 19.65	L: 169.93 ± 20.75	NS
Shoulder abduction (°)	R: 147.03 ± 9.56	R: 153.45 ± 13.39	R: 156.93 ± 17.12	NS
	L: 148.60 ± 13.71	L: 157.83 ± 15.94	L: 168.06 ± 22.25	NS
Trunk rotation (°)	R: 7.73 ± 2.69	**R: 6.25** **±** **6.25**	**R: 16.20** **±** **5.72**	0.021/**0.024**
	**L: 5.36** **±** **4.65**	L: 9.01 ± 4.96	**L: 13.46** **±** **4.01**	0.025/**0.023**
Trunk flexion/extension (°)	F: 30.11 ± 7.01	F: 32.11 ± 7.06	F: 44.78 ± 9.89	0.046
	E: 27.18 ± 7.04	E: 32.63 ± 18.91	E: 46.15 ± 7.35	0.049

Lunge distance (cm)	R: 62.11 ± 15.62	R: 70.49 ± 22.18	R: 85.11 ± 16.13	NS
	L: 58.52 ± 20.48	L: 68.52 ± 22.63	L: 83.15 ± 16.45	NS
*Gait items*				
Cadence (strides/min)	83.33 ± 14.21	90.38 ± 19.84	88.51 ± 4.62	NS
Stride length (cm)	97.28 ± 18.35	105.54 ± 11.66	107.07 ± 13.35	NS
Step length (cm)	R: 50.32 ± 9.65	R: 53.19 ± 7.76	R: 54.02 ± 7.80	NS
	L: 47.48 ± 8.90	L: 53.57 ± 4.49	L: 53.47 ± 5.38	NS
Step width (cm)	R: 10.01 ± 3.10	R: 10.08 ± 4.46	R: 10.13 ± 2.75	NS
	L: 8.78 ± 2.37	L: 7.76 ± 2.36	L: 8.46 ± 1.74	NS
Gait speed (m/s)	0.68 ± 0.24	0.81 ± 0.25	0.81 ± 0.09	NS

DBS, deep brain stimulation; NS, not significant; R, right; L, left; F, flexion; E, extension. ^*∗*^For right shoulder rotation, results are shown twice due to a double pairwise significant result.

**Table 4:** Statistically significant correlations between UPDRS-III and BME.

BME (UPDRS-III)^*∗*^	DBS Off		DBS On	
	SCC	*p* value	SCC	*p* value
Right shoulder flexion (UPDRS-III score)	−0.829	0.042	−0.486	NS
Right maximal abduction (UPDRS-III score)	−0.829	0.042	−0.029	NS
Right maximal abduction (upper limb rigidity)	−0.833	0.021	−0.169	NS
Bilateral squat depth (body bradykinesia)	−0.926	0.008	−0.676	NS
Bilateral squat depth (gait)	−0.926	0.008	−0.676	NS
Stride length (gait)	−0.833	0.039	−0.338	NS
Right step length (gait)	−0.926	0.008	−0.338	NS
Right step width (gait)	0.926	0.008	0.257	NS
Left step width (gait)	0.926	0.008	−0.167	NS
Cadence (arising from a chair)	−0.878	0.021	−0.676	NS
Stride length (arising from a chair)	−0.878	0.021	NA	NS
Gait speed (arising from a chair)	−0.878	0.021	NA	NS
Right trunk rotation (UPDRS-III score)	−0.086	NS	−0.928	0.008
Left trunk rotation (UPDRS-III score)	−0.257	NS	−0.829	0.048
Lunge distance, both sides (body bradykinesia)	−0.741	NS	−0.845	0.034
Lunge distance, both sides (gait)	−0.741	NS	−0.845	0.034
Cadence (posture)	−0.516	NS	−0.926	0.008
Gait speed (posture)	−0.638	NS	−0.926	0.008
Stride length (posture)	−0.577	NS	−0.833	0.039
Right step length (posture)	−0.395	NS	−0.833	0.039
Left step length (posture)	−0.698	NS	−0.833	0.039
Right shoulder flexion (UPDRS-III score)	−0.829	0.042	−0.486	NS
Right maximal abduction (UPDRS-III score)	−0.829	0.042	−0.029	NS
Right maximal abduction (upper limb rigidity)	−0.833	0.021	−0.169	NS
Two-leg squat displacement (body bradykinesia)	−0.926	0.008	−0.676	NS
Two-leg squat displacement (gait)	−0.926	0.008	−0.676	NS
Stride length (gait)	−0.833	0.039	−0.338	NS
Right step length (gait)	−0.926	0.008	−0.338	NS
Right step width (gait)	0.926	0.008	0.257	NS
Left step width (gait)	0.926	0.008	−0.167	NS
Cadence (rising from a chair)	−0.878	0.021	−0.676	NS
Stride length (rising from a chair)	−0.878	0.021	NA	NS
Gait speed (rising from a chair)	−0.878	0.021	NA	NS
Right trunk rotation (UPDRS-III score)	−0.086	NS	−0.928	0.008
Left trunk rotation (UPDRS-III score)	−0.257	NS	−0.829	0.048
Lunge displacement, both sides (body bradykinesia)	−0.741	NS	−0.845	0.034
Lunge displacement, both sides (gait)	−0.741	NS	−0.845	0.034
Cadence (posture)	−0.516	NS	−0.926	0.008
Gait speed (posture)	−0.638	NS	−0.926	0.008
Stride length (posture)	−0.577	NS	−0.833	0.039
Right step length (posture)	−0.395	NS	−0.833	0.039
Left step length (posture)	−0.698	NS	−0.833	0.039

^*∗*^Corresponding items to UPDRS-III. BME, body motion evaluation; DBS, deep brain stimulation; NA, not applicable because all data on “rising from a chair” were 0 in the On state; NS, not significant; SCC, Spearman correlation coefficient; UPDRS-III, Unified Parkinson's Disease Rating Scale.

## Data Availability

The data that support the findings of this study are available from the corresponding authors, MLG and HRM, at drhectormtz@yahoo.com and lucy.guerrero.gzz@gmail.com.
